# Increased body mass index is associated with operative difficulty during robot‐assisted radical prostatectomy

**DOI:** 10.1002/bco2.110

**Published:** 2021-09-27

**Authors:** Daniel D. Shapiro, John W. Davis, Wendell H. Williams, Brian F. Chapin, John F. Ward, Curtis A. Pettaway, Justin R. Gregg

**Affiliations:** ^1^ Department of Urology The University of Texas MD Anderson Cancer Center Houston Texas USA; ^2^ Department of Anesthesiology The University of Texas MD Anderson Cancer Center Houston Texas USA

**Keywords:** prostate cancer, prostatectomy, robotic surgery

## Abstract

**Objective:**

This study aimed to identify factors associated with surgeon perception of robot‐assisted radical prostatectomy (RARP) difficulty.

**Patients and Methods:**

This study surveyed surgeons performing RARP between 2017 and 2018 and asked them to rate operative conditions and difficulty as optimal, good, acceptable, or poor. These answers were stratified as optimal or suboptimal for this study. Associations between surgeon responses and variables hypothesized to affect surgical difficulty, including anatomic factors such as pelvic diameter and prostate volume:pelvic diameter ratio, were assessed.

**Results:**

Between November 2017 and September 2018, a total of 100 patients were prospectively enrolled in the study of which 58 cases were rated as optimal and 42 were rated as suboptimal. Of the evaluated variables, only increasing clinical T stage (odds ratio [OR] 1.49, 95% confidence interval [CI] 1.03–2.15, *p* = 0.03) and increasing body mass index (BMI) (OR 1.14, 95% CI 1.03–1.26, *p* = 0.01) were associated with increased difficulty; 90‐day complication rates were similar between the optimal and suboptimal cohorts (17.3% vs. 23.8%, respectively; *p* = 0.5). The number of patients with previous surgery, pelvic diameter, and prostate size:pelvic diameter ratio were not significantly different between cohorts (*p* > 0.05 for all). Operative time (*ρ* = 0.23, *p* = 0.02) and estimated blood loss (EBL) (*ρ* = 0.38, *p* = 0.0001) were correlated with suboptimal difficulty.

**Conclusion:**

The factors associated with surgeon‐reported RARP difficulty were patient BMI and clinical T stage among surgeons with significant RARP experience. These data should be incorporated into surgical decision making and patient counseling prior to performing a RARP.

## 1 INTRODUCTION

Robot‐assisted radical prostatectomy (RARP) is the most frequently used surgical approach in the United States for the management of clinically localized prostate cancer, with robotic utilization increasing rapidly from 1.8% of radical prostatectomies in 2003 to 85% in 2013.^1^ This technique has supplanted open techniques due to improvements in perioperative blood loss, length of stay, and surgeon ergonomics.^2^

Despite these technical advantages and widespread use, it is important for urologists and trainees to understand the factors that contribute to increased surgical complexity and difficulty during RARP. Technical challenges such as poor visibility, a small working space, increased intra‐abdominal fat, and obscure tissue planes may result in worse perioperative and postoperative outcomes; however, current studies have demonstrated conflicting results.^3^ Few studies have directly surveyed surgeons to identify challenging cases and what factors may influence the surgical complexity. Previous studies frequently use surrogate measures of surgical complexity such as estimated blood loss (EBL) or operative time, without directly evaluating surgeon feedback on the case complexity.^4^, ^5^, ^6^, ^7^, ^8^, ^9^

This study aimed to identify factors that are associated with a surgeon's perception of increased RARP difficulty. We hypothesized that anatomic factors such as body mass index (BMI) and the pelvic diameter would impact surgeon perception of difficulty. To conduct this study, we used standardized surveys administered during a randomized clinical trial to evaluate surgeon‐reported RARP difficulty.

## 2 PATIENTS AND METHODS

Patients enrolled in this study were part of an institutional review board‐approved randomized, double‐blind clinical trial to evaluate the effect of deep neuromuscular blockade with sugammadex reversal on shoulder pain of patients undergoing RARP at a single institution. The study is registered at https://www.clinicaltrials.gov/ (NCT03210376). The trial enrolled 100 patients, and as part of the trial, surgeons were asked to reduce insufflation pressure to the minimum level that allows for adequate visibility, which is consistent with how all surgeons routinely perform RARPs at this institution. At the end of each case, surgeons were given questionnaires and asked to evaluate the difficulty of the operation on an ordinal scale, selecting “optimal,” “good,” “acceptable,” or “poor” (Table S1).^10^ A total of seven surgeons participated in the study.

The present study tabulated the results of the surgeon rating and evaluated patient clinical and demographic factors that may have affected surgical difficulty. Variables included age, race, BMI, smoking history, prior abdominal or pelvic surgery, prior radiation therapy, preoperative systemic therapy, prostate volume, clinical T stage, pathologic Gleason grade group, pathologic TNM stage, extracapsular extension, and positive margin status. Few patients received preoperative systemic hormone ablation, which was not standardized and frequently started prior to the patients presenting at our institution. Most commonly, patients received preoperative systemic therapy for high‐risk or clinically node positive disease. Nerve sparing information was collected on the basis of operative notes and rated as “bilateral,” “partial or unilateral,” or “none.” Additionally, based on our hypothesis that pelvic diameter may be associated with perceived difficulty, the transverse pelvic diameter was calculated by using preoperative magnetic resonance imaging (MRI) imaging (which is routinely performed preoperatively at this institution) and measuring the transverse pelvic brim distance as previously described. This measurement was selected on the basis of previous studies looking at pelvic measurements to assess operative difficulty.^11^ We additionally created a prostate volume:pelvic diameter ratio as prostate size compared with diameter of the pelvis may impact the space available for robotic instruments and surgical difficulty. Prostate volume was calculated from preoperative MRI imaging measurements (prostate volume [cm^3^] = 0.52 × length × width × height).

A standard transabdominal RARP was performed by all surgeons in a similar fashion using carbon dioxide insufflation generally set at pressures of 12 mmHg or less. The insufflation pressure was recorded and averaged after each surgery. The performance of a lymph node dissection was at the discretion of each surgeon and was routinely performed for Grade Group 2 (GG2) or higher disease. An extended pelvic lymph node dissection template was most often utilized, which includes the area bounded by the external iliac vein anteriorly, pelvic sidewall laterally, floor of the pelvis posteriorly, Cooper's ligament distally, the bladder wall medially, and internal iliac artery proximally. Surgical variables were evaluated including duration of surgery, insufflation pressure, transfusion rates (including intraoperative and postoperative transfusions), and EBL. Intraoperative and 90‐day postoperative complications were recorded prospectively as part of the clinical trial design. Complications were graded according to the Clavien–Dindo classification.^12^ High‐grade complications were defined as Clavien IV or V.

Given the difficulty in determining the clinical significance of a surgeon rating a case as either acceptable or good and also data distribution, we chose to convert the initial rating scale into a dichotomous variable as either optimal (corresponding to the surgeon rating the case as “optimal”) or suboptimal (corresponding to the surgeon rating the case as “good,” “acceptable,” or “poor”). Demographic and clinical variables were compared between the optimal and suboptimal cohorts using Fisher's exact test for categorical variables and the Wilcoxon rank‐sum test for continuous variables. Univariable and multivariable logistic regression was used to determine predictors of difficulty, and Spearman correlation was used to determine correlations between difficulty and EBL or operative time surgical duration. Linear regression was used to evaluate the association between EBL and BMI and used to predict EBL across BMI. Statistical significance was considered if two‐tailed *p* value of <0.05. Statistical analysis was performed using Stata/SE Version 16.1 (StataCorp LP, College Station, TX).

## 3 RESULTS

A total of 100 patients were enrolled in the randomized clinical trial between November 2017 and September 2018. Table 1 demonstrates the clinical and pathologic characteristics of the patients divided into two cohorts based on the surgeon‐assessed operative difficulty. There were 58 surgeries rated as “optimal” compared with 42 surgeries rated as “suboptimal.” The variables noted to be significantly different between the optimal and suboptimal ratings included BMI and clinical T stage. The median BMI was higher in the suboptimal cohort compared with the optimal cohort (30.6 vs. 27.3 kg/m^2^, respectively, *p* = 0.004). Within the suboptimal cohort, more patients were noted to have cTstage > T1 compared with the optimal cohort (*p* = 0.03). No difference was noted in the rate of prior surgery, radiation, prostate volume, prostate volume:pelvic diameter ratio, or preoperative systemic therapy. Type of previous surgery was evaluated and compared between cohorts, and no significant difference was noted between cohorts based on surgery type (Table S2). Overall, there was no statistical difference in pathologic stage over all stages; however, there was a higher rate of stage pT3b in the suboptimal cohort compared with the optimal cohort (28.6% vs. 13.8%). The transverse pelvic brim distance was similar between the two cohorts. No difference was noted in predictors of recurrence such as the extracapsular extension rate (*p* = 0.7) or positive margin rate (*p* = 0.99) between the two cohorts.

**TABLE 1:** Clinical and pathologic characteristics

Variable	Overall (*N* = 100)	Optimal difficulty (*n* = 58)	Suboptimal difficulty (*n* = 42)	*p* value^a^
Median age (IQR)	68 (66.5–71)	68 (66–71)	68 (67–73)	0.7
Race, *n* (%)				0.7
Caucasian	78	47 (81)	31 (73.8)	
Black	9	5 (8.6)	4 (9.5)	
Hispanic	8	3 (5.2)	5 (11.9)	
Asian	5	3 (5.2)	2 (4.8)	
Median BMI (IQR)	28.6 (25.1–31.6)	27.3 (24.8–30.5)	30.6 (27.5–33.5)	0.004
Smoking history				0.9
Never	55	31 (53.5)	24 (57.1)	
Prior	39	23 (39.7)	16 (38.1)	
Current	6	4 (6.9)	2 (4.8)	
Prior abdominal or pelvic surgery	50	26 (44.8)	24 (57.1)	0.2
Prior radiation	1	1 (1.7)	0	1
Preoperative systemic therapy	13	7 (12.1)	6 (14.3)	0.8
Median prostate volume (IQR)	37.9 (24.1–55.2)	38.7 (26.1–62.6)	37.3 (21.7–47.2)	0.4
Clinical T stage				0.03
cT1	48 (83)	25 (59.5)		
cT2	8 (14)	12 (29)		
cT3	2 (3)	4 (9.5)		
cT4	0	1 (2)		
Median transverse pelvic diameter, cm (IQR)	12.3 (11.6–12.8)	12.1 (11.5–12.6)	12.4 (11.8–12.9)	0.2
Median prostate volume:pelvic diameter, cm^3^/cm (IQR)	3.17 (1.94–4.49)	3.25 (2.13–5.97)	2.91 (1.90–4.1)	0.4
Maximum pathologic tumor diameter, cm (IQR)	2 (1.5–3)	2 (1.5–2.6)	2.5 (2–3)	0.06
Nerve spare				0.3
Bilateral	60	35 (60.3)	25 (59.5)	
Unilateral/partial	20	14 (24.1)	6 (14.3)	
None	20	9 (15.5)	11 (26.2)	
Grade group				0.9
1	0	0	0	
2	44	27 (46.6)	17 (40.5)	
3	29	17 (29.3)	12 (28.6)	
4	2	1 (1.7)	1 (2.4)	
5	13	7 (12.1)	6 (14.3)	
Absent (due to preop hormone ablation)	12	6 (10.3)	6 (14.3)	
Pathologic T stage				0.2
pT2	52	32 (55.2)	20 (47.6)	
pT3a	28	18 (31)	10 (23.8)	
pT3b	20	8 (13.8)	12 (28.6)	
pT4	0	0	0	
N+	20	9 (15.5)	11 (26.2)	0.2
M+	3	2 (3.5)	1 (2.4)	0.3
Extracapsular extension	45	27 (46.6)	18 (42.9)	0.7
Positive margin	18	10 (17.2)	8 (19.1)	0.99

Abbreviations: BMI, body mass index; IQR, interquartile range.

^a^ Fisher's exact test or Wilcoxon rank‐sum test, when appropriate.

Surgical variables are listed in Table 2. The median length of surgery was longer in the cases rated as suboptimal versus optimal (median 207 vs. 172.5 min, respectively, *p* = 0.02). No difference was noted in the median insufflation pressure in the two cohorts. We evaluated the average insufflation pressure by BMI, and no significant association was demonstrated (*R* ^2^ = 0.4%, *p* = 0.5). The EBL was also higher in the suboptimal cohort (median 150 vs. 100 ml, *p* = 0.0002). Only two intraoperative complications were identified in the 100 patients, one occurring in each cohort.

**TABLE 2:** Surgical variables

Variable	Overall cohort (*N* = 100)	Optimal difficulty (*n* = 58)	Suboptimal difficulty (*n* = 42)	*p* value
Median operative time, min (IQR)	187.5 (156.5–226)	172.5 (149–215)	207 (170–239)	0.02
Median insufflation pressure (IQR)	12.1 (10.5–12.9)	12.2 (10.4–13.1)	12.1 (10.8–12.8)	0.9
Median estimated blood loss, ml (IQR)	100 (100–175)	100 (100–150)	150 (100–250)	0.0002
Surgeon difficulty rating				
Poor	2	0	2 (4.8)	
Acceptable	13	0	13 (31)	
Good	27	0	27 (64.3)	
Optimal	58	58 (100)	0	
Intraoperative complication, *n* (%)	2	1 (1.7)	1 (2.4)	0.99

Abbreviation: IQR, interquartile range.

Table 3 lists the postoperative outcomes by cohort. Transfusion (either intraoperative or postoperatively) was a rare event overall, occurring once in each cohort. No difference was noted in the 90‐day complication rate, with 10 complications occurring in each cohort (*p* = 0.5). Only one high‐grade postoperative complication occurred overall and occurred in the suboptimal difficulty cohort in which a patient developed sepsis and abscess formation requiring intensive care unit (ICU) admission and drain placement. The 30‐day readmission rate was higher in the optimal cohort (10.3% vs. 0%, *p* = 0.04). The reasons for readmission were ileus for four patients, non‐ST‐elevation myocardial infarction for one patient, and catheter replacement requiring flexible cystoscopy for one patient.

**TABLE 3:** Postoperative outcomes

Variable	Overall cohort (*N* = 100)	Optimal difficulty (*N* = 58)	Suboptimal difficulty (*N* = 42)	*p* value
Transfusion	2	1 (1.7)	1 (2.4)	0.7
30‐day readmission	6	6 (10.3)	0	0.04
90‐day postoperative complication	20	10 (17.3)	10 (23.8)	0.5
Clavien–Dindo classification				0.5
I	5	2 (3.5)	3 (7.1)	
II	7	3 (5.2)	4 (9.5)	
IIIa	4	2 (3.5)	2 (4.8)	
IIIb	3	3 (5.2)	0	
IVb	1	0	1 (2.4)	
V	0	0	0	

**FIGURE 1:**
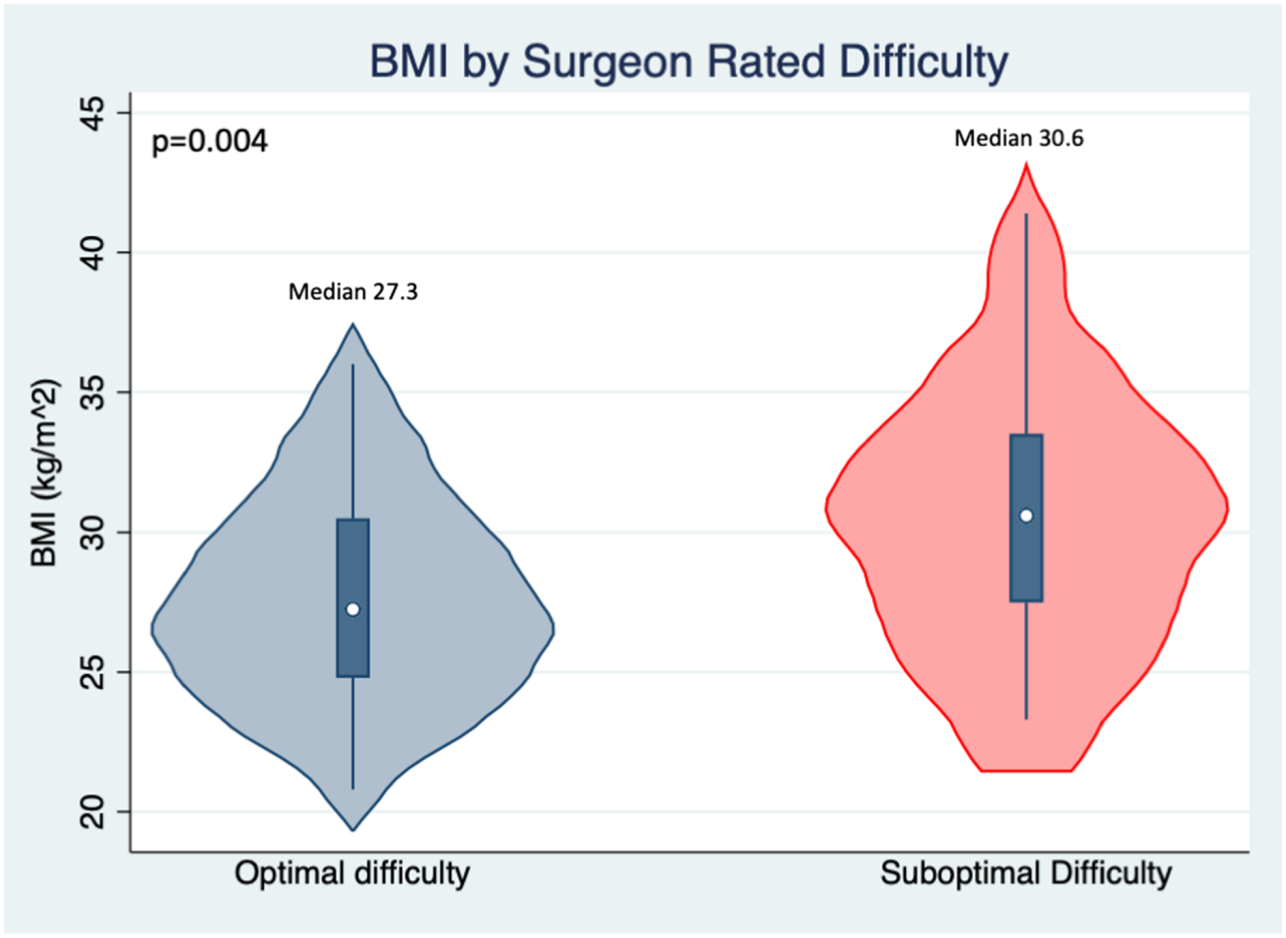
Violin plots of body mass index (BMI) by surgeon‐assessed robot‐assisted radical prostatectomy difficulty cohort

Univariable and multivariable logistic regression was performed to evaluate associations between clinical variables and surgeon‐assessed difficulty. After univariable analysis, the only two predictors of increased difficulty were BMI (odds ratio [OR] 3.18, 95% confidence interval [CI] 1.38–7.94, *p* = 0.007) and increasing clinical T stage (OR 1.64, 95% CI 1.14–2.36, *p* = 0.008). BMI was additionally included as a continuous variable, which was also significantly associated with surgeon‐assessed difficulty (OR 1.17, 95% CI 1.06–1.30, *p* = 0.003). To ensure BMI was not acting as a confounding variable for clinical T stage, a multivariable model including both BMI and clinical T stage demonstrated that BMI (*p* = 0.01) and clinical T stage (*p* = 0.03) were both independently associated with surgeon‐assessed difficulty (Table 4). Figure 1 demonstrates the distribution violin plot of the BMI stratified by difficulty cohort, demonstrating the higher range of BMI in the suboptimal cohort. Increased BMI was also associated with increased predicted EBL (Figure S1) (*R* ^2^ = 7.5%, *p* = 0.006).

**TABLE 4:** Associations with clinical variables and surgeon‐assessed difficulty

Variable	Univariable			Multivariable		
	OR	95% CI	*p* value	OR	95% CI	*p* value
Age	1.02	0.92–1.14	0.69			
BMI (continuous)	1.17	1.06–1.30	0.003	1.14	1.03–1.26	0.01
BMI (≥30 vs. <30)	3.18	1.38–7.94	0.007			
MRI prostate volume	0.99	0.97–1.01	0.36			
Transverse pelvic diameter	1.12	0.68–1.83	0.66			
Prostate volume:pelvic diameter	0.89	0.71–1.12	0.31			
Prior abdominal surgery	1.64	0.74–3.66	0.23			
Smoking history	0.86	0.39–1.92	0.71			
Prior systemic therapy	1.21	0.38–3.92	0.75			
Clinical T stage	1.64	1.14–2.36	0.008	1.49	1.03–2.15	0.03
Nerve sparing						
Bilateral	Ref					
Partial/unilateral	0.6	0.20–1.78	0.4			
None	1.71	0.62–4.74	0.3			

Abbreviations: BMI, body mass index; CI, confidence interval; MRI, magnetic resonance imaging; OR, odds ratio.

Lastly, we sought to determine the correlation between EBL and operative time and surgeon‐assessed difficulty. Both EBL (*ρ* = 0.38, *p* = 0.0001) and operative time (*ρ* = 0.23, *p* = 0.02) were significantly associated with increased difficulty (Table S3).

## 4 DISCUSSION

Understanding the factors that impact case complexity and how complexity relates to surgical outcomes and cost helps surgeons to select appropriate patients for surgery, counsel patients prior to surgery, and effectively communicate with members of the surgical team perioperatively. Additionally, less experienced surgeons can use factors associated with increased surgical difficulty to identify cases that may require additional assistance or time based on the surgeon's current technical proficiency. Using prospectively collected surveys of surgeon‐assessed RARP difficulty, this study demonstrated that the main predictors of increased surgical difficulty were increasing patient BMI and increasing clinical T stage.

The knowledge of a more advanced T stage, which can be identified on preoperative imaging or digital rectal exam, may require surgeons to perform a wide dissection in the area around the concerning tumor, increasing the surgical difficulty. Extracapsular tumor extension posteriorly often requires resection of Denonvilliers' fascia and close dissection along the rectum.^13^ Additionally, a more extensive lymph node dissection may be performed for locally advanced tumors.^14^

Although previous studies have evaluated the relationship between obesity and postoperative complications,^15^ few have directly evaluated the effect of obesity on surgeon‐assessed difficulty. Han et al. used the National Inpatient Sample (NIS) database to evaluate the effects of morbid obesity on perioperative outcomes, demonstrating a 17% postoperative complication rate among the morbidly obese compared with 7.6% for nonobese patients. Additionally, morbid obesity was statistically associated with an increased number of postoperative complications after propensity score matching.^16^ Within our study, we demonstrated that increased BMI is associated with an increase in EBL, which is supported by a study by Sundi et al. showing obesity to be significantly associated with increased EBL and operative time.^17^ Although the study by Sundi et al. did not directly measure surgical difficulty, they hypothesized increased EBL and operative time to be indicative of surgical difficulty.^17^ Several factors have been attributed to the technical challenges when performing RARP on obese patients. These include limited working space leading to poor visualization, increased distance from the skin to the working site, positioning, and ventilation issues when patients are placed in steep Trendelenburg.^17^, ^18^ Understanding the difficulties associated with operating on obese patients is important as obesity has been previously associated with not only increasing postoperative complications but also worse measures of postoperative quality of life.^15^, ^18^, ^19^

In the current literature, operative time and EBL are often used as surrogate markers for surgical difficulty without directly assessing surgeon‐reported difficulty.^4^, ^5^, ^6^, ^7^, ^8^, ^9^ We demonstrate that EBL and operative time are associated with surgeon‐reported difficulty, and EBL and operative time increased in the suboptimal cohort. Although we did not see an increase in complication or readmission rate among the suboptimal cohort, increasing operative time and EBL are associated with increasing cost. A study by Peard et al. demonstrated that among patients undergoing robot‐assisted laparoscopic radical prostatectomy (RALP), increasing operative time and EBL were associated with increased direct and total costs.^20^

Previous studies of laparoscopic and robotic surgery have demonstrated that pelvic measurements may impact the difficulty of pelvic surgery due to limited working space.^4^, ^5^, ^6^, ^7^, ^11^, ^21^ Using measurements including transverse pelvic diameter as well as the prostate volume:pelvic diameter ratio, we found no significant increase in surgeon‐assessed difficulty. This may be due to surgeon experience, as Yao et al. found that the association between prostate size and pelvic dimensions was no longer a predictor of increased EBL as surgeons gained more experience.^7^ Additionally, the size differences in pelvic dimensions measured in this study were similar between patients, which may limit the ability to find a statistical difference between the optimal and suboptimal cohorts. The findings of our study are supported by a study from Hong et al. that demonstrated that among a cohort of men undergoing RARP, pelvic dimensions were not associated with operative duration or EBL.^22^

About half of the patients in both the optimal and suboptimal cohorts had undergone a prior abdominal or pelvic surgery (which included endoscopic transurethral surgeries), suggesting a limited influence of prior surgery on RARP difficulty. Some prior studies have demonstrated an increased difficulty among patients who have had prior genitourinary or abdominal surgery.^23^, ^24^, ^25^ Patient selection and surgeon experience likely play a significant role in whether prior surgery impacts surgeon‐reported difficulty. Patients with complex prior abdominal surgery are likely directed to other treatment modalities such as radiation therapy. Patient selection was likely a primary factor limiting the association between surgical history and surgeon‐assessed difficulty in this study.

Within this study, we found a high‐grade (Clavien Grade IV–V) postoperative complication rate of 1%. This is consistent with previous studies that report the majority of complications being low‐grade (Clavien ≤III) and high‐grade complication rates of 1–3%.^26^, ^27^, ^28^ The presence of high‐grade complications is often influenced by the performance of a lymph node dissection. At our institution, we routinely perform lymph node dissections for all patients with GG2 or higher prostate cancer, and all patients within this study were GG ≥ 2. Given that all patients within this study had a lymph node dissection, this could not be included as a variable associated with surgeon‐reported difficulty. We did not find that surgeon‐reported difficulty to be strongly associated with rates of overall complications. Notably, we found that readmissions were more common among the optimal cohort. These data suggest that other patient‐related factors such as comorbidities are likely more important factors affecting postoperative complication and readmissions than the technical difficulty of a surgery.

This study is strengthened by prospectively assessing surgeon‐reported case difficulty using a standardized questionnaire. This study, however, has several limitations. The study sample size is relatively small. All surgeons assessed using the questionnaire have significant experience performing RARPs at a single high‐volume tertiary care center, which may limit the generalizability of the findings to other populations. This may also explain the lack of a significant difference in complications between the optimal and suboptimal cohorts, even among patients with an elevated BMI. Previous reports demonstrate that patients undergoing RARP at high‐volume centers have improved postoperative outcomes and reduced complications.^29^ The surgeons in this study have all been in practice for ≥8 years, and the factors influencing perceived surgical difficulty may be different for surgeons assessed earlier in their learning curve. Additional, unmeasured factors that impact surgical difficulty may be present that were not addressed in this study. Lastly, the specific reasons why surgeons rated a RARP as less than optimal were not assessed in this series, and the survey instrument used, although intuitive, has not been previously validated.

## 5 CONCLUSION

In summary, the primary factors associated with surgeon‐assessed RARP difficulty were patient BMI and clinical T stage among surgeons with significant RARP experience. Although likely not surprising to surgeons performing RARP, these data should be incorporated into surgical decision making and patient counseling prior to performing a RARP.

## CONFLICT OF INTERESTS

J. W. Davis reports consulting for Intuitive Surgical and research support from GenomeDX and Janssen Pharmaceuticals. B. F. Chapin reports consulting for Blue Earth Diagnostics and research funding from Janssen Pharmaceuticals. The remaining authors have no disclosures.

## AUTHOR CONTRIBUTION

Daniel D. Shapiro, Justin R. Gregg ‐ conception, analysis, data collection, manuscript creation. John W. Davis, Wendell H. Williams, Brian F. Chapin, John F. Ward, Curtis A. Pettaway ‐ recruitment, study conception, editing.

## Supporting information


**Table S1.** Survey distributed to surgeons to assess the robotic assisted radical prostatectomy case conditions and difficulty.
**Table S2.** Prior surgery stratified by cohort.
**Table S3.** Spearman correlation between surgeon assessed RARP difficulty and estimated blood loss (EBL) or operative time.

**Figure S1.** Predicted estimated blood loss (EBL) by body mass index (BMI) determined by linear regression. Regression equation is displayed in the bottom right corner of the figure.

## References

[1] Leow JJ , Chang SL , Meyer CP , Wang Y , Hanske J , Sammon JD , et al. Robot‐assisted versus open radical prostatectomy: a contemporary analysis of an all‐payer discharge database. Eur Urol. 2016;70(5):837–45. 26874806 10.1016/j.eururo.2016.01.044

[2] Seo HJ , Lee NR , Son SK , Kim DK , Rha KH , Lee SH . Comparison of robot‐assisted radical prostatectomy and open radical prostatectomy outcomes: a systematic review and meta‐analysis. Yonsei Med J. 2016;57(5):1165–77. 27401648 10.3349/ymj.2016.57.5.1165 PMC4960383

[3] Erestam S , Bock D , Erichsen Andersson A , Bjartell A , Carlsson S , Stinesen Kollberg K , et al. Associations between intraoperative factors and surgeons' self‐assessed operative satisfaction. Surg Endosc. 2020;34(1):61–8. 30887183 10.1007/s00464-019-06731-z PMC6946718

[4] Li Q , Li D , Jiang L , Qiu P , Fu Z , Tang L , et al. Factors influencing difficulty of laparoscopic abdominoperineal resection for ultra‐low rectal cancer. Surg Laparosc Endosc Percutan Tech. 2017;27(2):104–9. 28212258 10.1097/SLE.0000000000000378 PMC5378004

[5] Escal L , Nougaret S , Guiu B , Bertrand MM , de Forges H , Tetreau R , et al. MRI‐based score to predict surgical difficulty in patients with rectal cancer. Br J Surg. 2018;105(1):140–6. 29088504 10.1002/bjs.10642

[6] Mason BM , Hakimi AA , Faleck D , Chernyak V , Rozenblitt A , Ghavamian R . The role of preoperative endo‐rectal coil magnetic resonance imaging in predicting surgical difficulty for robotic prostatectomy. Urology. 2010;76(5):1130–5. 20739048 10.1016/j.urology.2010.05.037

[7] Yao A , Iwamoto H , Masago T , Morizane S , Honda M , Sejima T , et al. Anatomical dimensions using preoperative magnetic resonance imaging: impact on the learning curve of robot‐assisted laparoscopic prostatectomy. Int J Urol. 2015;22(1):74–9. 25212691 10.1111/iju.12602

[8] Horuz R , Göktaş C , Çetinel CA , Akça O , Cangüven Ö , Şahin C , et al. Simple preoperative parameters to assess technical difficulty during a radical perineal prostatectomy. Int Urol Nephrol. 2013;45(1):129–33. 23054325 10.1007/s11255-012-0310-1

[9] Pettus JA , Masterson T , Sokol A , Cronin AM , Savage C , Sandhu JS , et al. Prostate size is associated with surgical difficulty but not functional outcome at 1 year after radical prostatectomy. J Urol. 2009;182(3):949–55. 19616260 10.1016/j.juro.2009.05.029 PMC2885288

[10] Williams WH III , Cata JP , Lasala JD , Navai N , Feng L , Gottumukkala V . Effect of reversal of deep neuromuscular block with sugammadex or moderate block by neostigmine on shoulder pain in elderly patients undergoing robotic prostatectomy. Br J Anaesth. 2020;124(2):164–72. 31780139 10.1016/j.bja.2019.09.043

[11] Neill MG , Lockwood GA , McCluskey SA , Fleshner NE . Preoperative evaluation of the ‘hostile pelvis’ in radical prostatectomy with computed tomographic pelvimetry. BJU Int. 2007;99(3):534–8. 17155982 10.1111/j.1464-410X.2006.06640.x

[12] Dindo D , Demartines N , Clavien PA . Classification of surgical complications: a new proposal with evaluation in a cohort of 6336 patients and results of a survey. Ann Surg. 2004;240(2):205–13. 15273542 10.1097/01.sla.0000133083.54934.ae PMC1360123

[13] Mazzone E , Dell'Oglio P , Rosiello G , Puliatti S , Brook N , Turri F , et al. Technical refinements in superextended robot‐assisted radical prostatectomy for locally advanced prostate cancer patients at multiparametric magnetic resonance imaging. Eur Urol. 2020;80(1):104–12. 32943260 10.1016/j.eururo.2020.09.009

[14] Fossati N , Willemse PM , Van den Broeck T , van den Bergh RCN , Yuan CY , Briers E , et al. The benefits and harms of different extents of lymph node dissection during radical prostatectomy for prostate cancer: a systematic review. Eur Urol. 2017;72(1):84–109. 28126351 10.1016/j.eururo.2016.12.003

[15] Murphy DG , Bjartell A , Ficarra V , Graefen M , Haese A , Montironi R , et al. Downsides of robot‐assisted laparoscopic radical prostatectomy: limitations and complications. Eur Urol. 2010;57(5):735–46. 20036784 10.1016/j.eururo.2009.12.021

[16] Han H , Qin Y , Ruan Y , He J , Cao Z , Wei X , et al. Morbid obesity is adversely associated with perioperative outcomes in patients undergoing robot‐assisted laparoscopic radical prostatectomy. Can Urol Assoc J. 2020. 10.5489/cuaj.6389 PMC7673829 32520702

[17] Sundi D , Reese AC , Mettee LZ , Trock BJ , Pavlovich CP . Laparoscopic and robotic radical prostatectomy outcomes in obese and extremely obese men. Urology. 2013;82(3):600–5. 23859532 10.1016/j.urology.2013.05.013 PMC3758791

[18] Samavedi S , Abdul‐Muhsin H , Pigilam S , Sivaraman A , Patel VR . Handling difficult anastomosis. Tips and tricks in obese patients and narrow pelvis. Indian J Urol. 2014;30(4):418–22. 25378824 10.4103/0970-1591.142070 PMC4220382

[19] Tang K , Jiang K , Chen H , Chen Z , Xu H , Ye Z . Robotic vs. retropubic radical prostatectomy in prostate cancer: a systematic review and a meta‐analysis update. Oncotarget. 2017;8(19):32237–57. 27852051 10.18632/oncotarget.13332 PMC5458281

[20] Peard L , Goodwin J , Hensley P , Dugan A , Bylund J , Harris AM . Examining and understanding value: the impact of preoperative characteristics, intraoperative variables, and postoperative complications on cost of robot‐assisted laparoscopic radical prostatectomy. J Endourol. 2019;33(7):541–8. 31017013 10.1089/end.2019.0066

[21] Ogiso S , Yamaguchi T , Hata H , Fukuda M , Ikai I , Yamato T , et al. Evaluation of factors affecting the difficulty of laparoscopic anterior resection for rectal cancer: “narrow pelvis” is not a contraindication. Surg Endosc. 2011;25(6):1907–12. 21136101 10.1007/s00464-010-1485-0

[22] Hong SK , Lee ST , Kim SS , Min KE , Hwang IS , Kim M , et al. Effect of bony pelvic dimensions measured by preoperative magnetic resonance imaging on performing robot‐assisted laparoscopic prostatectomy. BJU Int. 2009;104(5):664–8. 19466945 10.1111/j.1464-410X.2009.08624.x

[23] Gupta NP , Singh P , Nayyar R . Outcomes of robot‐assisted radical prostatectomy in men with previous transurethral resection of prostate. BJU Int. 2011;108(9):1501–5. 21392223 10.1111/j.1464-410X.2011.10113.x

[24] Goldstraw MA , Challacombe BJ , Patil K , Amoroso P , Dasgupta P , Kirby RS . Overcoming the challenges of robot‐assisted radical prostatectomy. Prostate Cancer Prostatic Dis. 2012;15(1):1–7. 21844888 10.1038/pcan.2011.37

[25] Yazici S , Inci K , Yuksel S , Bilen CY , Ozen H . Radical prostatectomy after previous prostate surgery: effects on surgical difficulty and pathologic outcomes. Urology. 2009;73(4):856–9. 19022487 10.1016/j.urology.2008.09.024

[26] Pompe RS , Beyer B , Haese A , Preisser F , Michl U , Steuber T , et al. Postoperative complications of contemporary open and robot‐assisted laparoscopic radical prostatectomy using standardised reporting systems. BJU Int. 2018;122(5):801–7. 29727912 10.1111/bju.14369

[27] Yaxley JW , Coughlin GD , Chambers SK , Occhipinti S , Samaratunga H , Zajdlewicz L , et al. Robot‐assisted laparoscopic prostatectomy versus open radical retropubic prostatectomy: early outcomes from a randomised controlled phase 3 study. Lancet. 2016;388(10049):1057–66. 27474375 10.1016/S0140-6736(16)30592-X

[28] Gandaglia G , De Lorenzis E , Novara G , Fossati N , De Groote R , Dovey Z , et al. Robot‐assisted radical prostatectomy and extended pelvic lymph node dissection in patients with locally‐advanced prostate cancer. Eur Urol. 2017;71(2):249–56. 27209538 10.1016/j.eururo.2016.05.008

[29] Xia L , Sperling CD , Taylor BL , Talwar R , Chelluri RR , Raman JD , et al. Associations between hospital volume and outcomes of robot‐assisted radical prostatectomy. J Urol. 2020;203(5):926–32. 31846391 10.1097/JU.0000000000000698

