# Supplementary material for: Increased body mass index is associated with operative difficulty during robot‐assisted radical prostatectomy
Source: BJUI Compass. 2021 Sep 27;3(1):68–74. doi: 10.1002/bco2.110 (PMC8988518; doi:10.1002/bco2.110)
Supplement: Supplementary file 1 — Table S1. Survey distributed to surgeons to assess the robotic assisted radical prostatectomy case conditions and difficulty. Table S2. Prior surgery stratified by cohort. Table S3. Spearman correlation between surgeon assessed RARP difficulty and estimated blood loss (EBL) or operative time. [file BCO2-3-68-s002.docx]

**Table S1.** Survey distributed to surgeons to assess the robotic assisted radical prostatectomy case conditions and difficulty.

| **How would you rate the surgical conditions and difficulty of the case?** | |
| --- | --- |
| Optimal |  |
| Good |  |
| Acceptable |  |
| Poor |  |

**Table S2.** Prior surgery stratified by cohort.

| **Prior Surgery** | **Overall Cohort (N=100)** | **Optimal difficulty**  **(N=58)** | **Suboptimal difficulty (N=42)** | **p-value** |
| --- | --- | --- | --- | --- |
| Abdominal (non-hernia) | 35 | 18 (31) | 17 (40.5) | 0.4 |
| Hernia repair | 18 | 10 (17.2) | 8 (19.1) | 0.99 |
| Transurethral prostate intervention | 10 | 6 (10.3) | 4 (9.5) | 0.99 |
| Any surgery (abdominal, hernia or transurethral)* | 50 | 26 (44.8) | 24 (57.1) | 0.3 |

*Some patients may have had more than one type of prior surgery

**Table S3.** Spearman correlation between surgeon assessed RARP difficulty and estimated blood loss (EBL) or operative time.

| **Spearman correlation between difficulty and EBL or surgical time** | **rho** | **p-value** |
| --- | --- | --- |
| EBL | 0.38 | 0.0001 |
| Operative time | 0.23 | 0.02 |
