# Supplementary figures and images for: Increased body mass index is associated with operative difficulty during robot‐assisted radical prostatectomy
Source: BJUI Compass. 2021 Sep 27;3(1):68–74. doi: 10.1002/bco2.110 (PMC8988518; doi:10.1002/bco2.110)

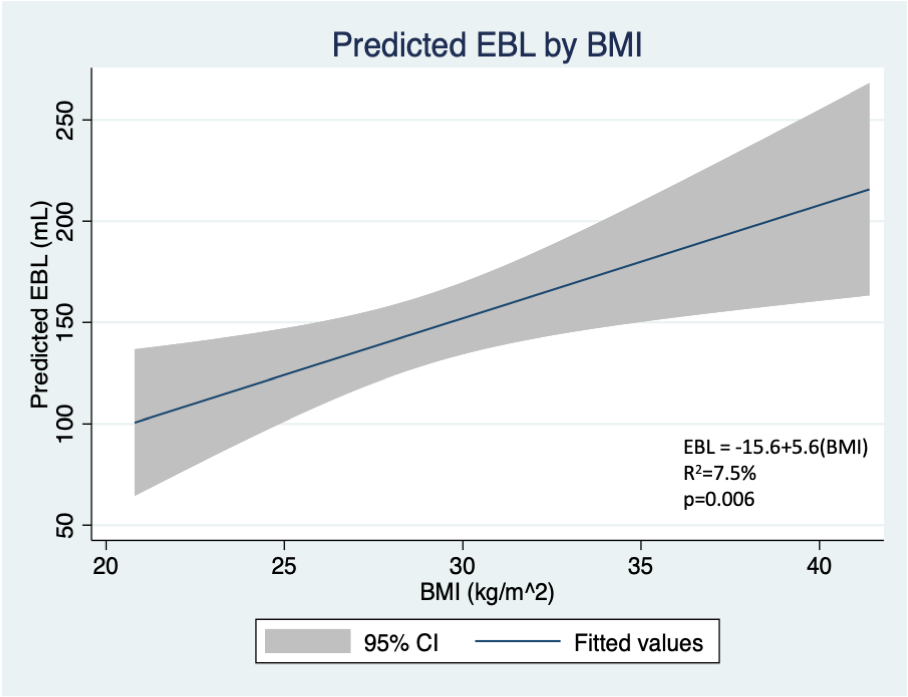

Supplement: Supplementary file 2 — Figure S1. Predicted estimated blood loss (EBL) by body mass index (BMI) determined by linear regression. Regression equation is displayed in the bottom right corner of the figure. [file BCO2-3-68-s001.png]
